# Pemetrexed-induced acute kidney failure following irreversible renal damage: two case reports and literature review

**DOI:** 10.15171/jnp.2017.07

**Published:** 2016-10-27

**Authors:** Tito Zattera, Francesco Londrino, Matteo Trezzi, Roberto Palumbo, Antonio Granata, Paola Tatangelo, Valentina Corbani, Valeria Falqui, Nadia Chiappini, Lisa Mathiasen, Marco Cavallini, Davide Rolla

**Affiliations:** ^1^Unità Operativa Complessa, Nefrologia e Dialisi, Ospedale S. Andrea, La Spezia, Italy; ^2^Unità Operativa Complessa, Nefrologia e Dialisi, Ospedale S. Eugenio, Roma, Italy; ^3^Unità Operativa Complessa, Nefrologia e Dialisi, Ospedale S. Giovanni di Dio, Agrigento, Italy; ^4^Estor, Pero, Italy; ^5^Unità Operativa Complessa, Nefrologia e Dialisi, Ospedale di Dolo, Italy

**Keywords:** Pemetrexed, Acute kidney injury, Nephrotoxicity

## Abstract

**Background:**

Pemetrexed (PEM) is a new-generation multitargeted antifolate agent with a demonstrated broad-spectrum activity in several types of human cancers, including non-small cell lung cancer (NSCLC) and mesothelioma. Major side effects include dose-limiting hematologic toxicities. PEM nephrotoxicity is well known; however, its frequency is considered to be low.

**Case Presentation:**

Here we report two cases of acute kidney injury (AKI) related to PEM administration (500 mg/m2) in patients with NSCLC. The first patient required hemodialysis treatment and was submitted to renal biopsy which showed acute tubular damage and interstitial edema without acute tubular necrosis. No other potential nephrotoxic agents were identified. The second patient developed AKI, not proven by biopsy and did not require renal replacement therapy. Both patients, on regular supplementation with folic acid and vitamin B12, concomitantly developed myelosuppression and even several months after PEM withdrawal, showed only a modest improvement of renal function.

**Conclusions:**

PEM is an antifolate antineoplastic agent with a broad-spectrum activity in locally advanced or metastatic NSCLC. It has been shown that PEM allows longer survival. The risk of acute or chronic kidney disease may be one of the prices to be paid for this success.

**Implication for health policy/practice/research/medical education:**

Pemetrexed (PEM) is an antifolate antineoplastic agent with a broad-spectrum activity in locally advanced or metastatic non-small cell lung cancer (NSCLC). It has been shown that PEM allows longer survival. The risk of acute or chronic kidney disease may be one of the prices to be paid for this success. This paper demonstrates that nephrologists and oncologists should collaborate to study tailored therapies in patients with cancer.

## 1. Introduction

Pemetrexed (PEM) is an antifolate antineoplastic agent which has shown clinical efficacy in patients with locally advanced or metastatic non-small cell lung cancer (NSCLC) and mesothelioma. It has been demonstrated, that PEM acts as a thymidylate synthase inhibitor, interrupting DNA synthesis, which results in decreased cell growth and induced apoptosis (1). PEM can be used alone or in combination with other antineoplastic drugs, such as cisplatin. The main adverse effects of PEM include myelosuppression and hepatotoxicity, which may be prevented by folic acid supplementation. Other toxicities include rash, mucositis, nausea, and vomiting (2-5). PEM nephrotoxicity is well known; however, its frequency is considered to be low. Physiopathological mechanisms of renal damage remain unknown. International literature reports cases of acute kidney injury (AKI) with or without tubular necrosis, distal tubular acidosis or interstitial nephritis, and in a few cases AKI was associated with diabetes insipidus. Only few patients were submitted to renal biopsy.

We report two cases of AKI with only a partial recovery of renal function following PEM therapy in two patients with NSCLC.

## 2. Case Presentation

### 2.1. Case 1

The patient was a male, aged 52 years, affected by pulmonary adenocarcinoma with multiple bone metastases (stage IV), diagnosed in June 2009.

The patient had undergone first line chemotherapy with cisplatin (75 mg/m^2^, day 1 q 21) and gemcitabine (1250 mg/m^2^, day 1-8 q 21) for five cycles from July to November 2009. In April 2010, due to significant worsening of skeletal involvement, a second line therapy was performed, involving six cycles of PEM (500 mg/m^2^, day 1 q 21). Vitamin B12, folic acid and steroids were also administered. Sporadic consumption of non-steroidal anti-inflammatory drugs (NSAIDs) was reported.

On August 17, 2010, serum creatinine was 1 mg/dL (glomerular filtration rate; GFR: 86 mL/min/1.73 m^2^, CKD-EPI), but then it increased to 2.2 mg/dL. In September 2010, after the seventh cycle of PEM, creatinine value was 4 mg/dL. No other nephrotoxic agents were identified.

Thus, the patient was admitted in our department. There were a severe reduction of kidney function (serum creatinine 5 mg/dL), megaloblastic anemia, modest metabolic acidosis (pH 7.31, pHCO_3_ 21 mM) and modest hyperkalemia (pK 5.5 mM). Diabetes was excluded. Urine analysis showed proteinuria (~500 mg/dL). No sediment alteration was detected. From the ultrasound images, both kidneys appeared enlarged (14 cm) and swollen, indicative of AKI.

Intra-parenchymal resistive indices (RIs) were at the upper limits (right RI: 0.73, left RI: 0.72) and a kidney biopsy was performed.

**Figure 1:**
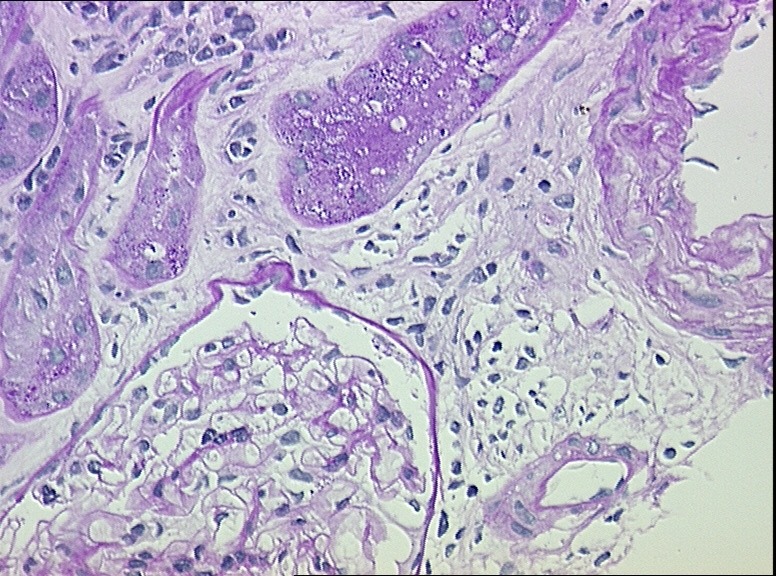
Light microscopy image showing part of one glomerulus with no change, interstitial edema with slight mononuclear cells infiltrate, the central tubule with disappearance of the lumen and vacuolation and swelling of the cytoplasm. PAS stain, x400.

**Figure 2:**
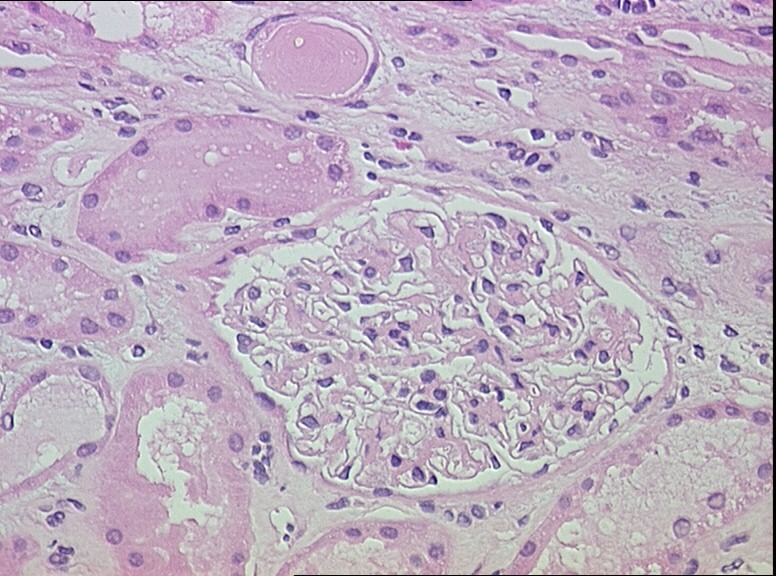
Light microscopy image evidencing a normal glomerulus, slight interstitial edema, swelling and vacuolation of the proximal convoluted tubules. Hematoxylin and Eosin stain, x200.

The renal biopsy evaluated with light microscopy, showed six glomeruli with only minor changes, such as the presence of one obsolescent glomerulus, slight interstitial edema, swelling and vacuolation of many convoluted proximal tubules (Figures 1 and 2). The immunofluorescent study was negative. Normal glomerular ultrastructure was confirmed by electron microscopy. The proximal convoluted tubules showed mild swelling and vacuolation of the cytoplasm.

One week later hemodialysis was started due to further deterioration of kidney function (creatinine 6.7 mg/dL). Laboratory and renal sonographic follow-up were performed for five months and dialysis was suspended when the patient experienced partial recovery of renal function (creatinine 4.1 mg/dL, GFR 16 mL/min). Ultrasound images showed less enlarged kidneys, but also a progressive thinning of renal cortex and a reduction in cortico-medullary differentiation. RIs remained elevated. The patient’s creatinine level remained elevated, but stable.

### 2.2. Case 2

In July 2008, the patient, a male aged 56 years, underwent a left inferior lung lobectomy (pT1N1M0), owing to a left pulmonary adenocarcinoma. Three cycles of chemotherapy with carboplatin (AUC = 5 mg/mL/min.) and paclitaxel (175 mg/m^2^) (1 q 21) were performed until September 2008. In March 2009, the patient underwent a right adrenalectomy to treat a metastatic lesion. In May 2009, nuclear magnetic resonance (NMR) imaging showed a left adrenal metastasis, which was confirmed by positron emission tomography (PET)/computerized tomography (CT). Therefore, the patient entered a study protocol of second line chemotherapy, which included PEM (500 mg/m^2^, 1 q 21), carboplatin (AUC = 5 mg/mL/min.) and paclitaxel (175 mg/m^2^) (1 q 21). Adequate premedication with folic acid and vitamin B12 was administered. Serum creatinine, prior to therapy, was 1.2 mg/dL, (glomerular filtration rate [GFR] 67 mL/min/1.73 m^2^, CKD-EPI). There were no alterations of urine analysis. The total body CT performed in September showed a significant reduction of the secondary lesions.

One month later, during the sixth cycle with PEM, the patient was admitted into the department of medicine due to acute, non-oliguric renal impairment, vomiting and pancytopenia. Although hypovolemia was corrected, creatinine remained elevated (4 mg/dL). The patient was then transferred to our unit. Unfortunately, renal function continued to decline although euvolemia was maintained and blood pressure was controlled. Proteinuria was present (400 mg/dL), sediment was negative as well as all other immunological tests and serum immunofixation.

At ultrasound examination, both kidneys appeared enlarged. Intra-parenchymal-RIs were augmented (RI: 0.75).

Considering the absence of urine analysis alterations, the negativity of immunological tests and, the intrinsic characteristics of the AKI, we decided not to perform a kidney biopsy. The patient was then discharged and continued follow-up.

In December 2009, creatinine was 3 mg/dL, GFR was 22 mL/min/1.73 m^2^. Subsequent ambulatory controls confirmed these data.

## 3. Discussion

PEM (Alimta^®^) is an antifolate antineoplastic agent effective in several tumor types, such as NSCLC and mesothelioma, among others. Recommended dose is 500 mg/m^2^ intravenous as a single agent or in combination with cisplatin, on a once every 21 day schedule. The drug is not metabolized significantly. About 80% is completely eliminated unchanged by the renal emunctory, and therefore its use is contraindicated if creatinine clearance is below 45 mL/min. Up to 80% of the drug is bound to plasma proteins, the apparent distribution volume is 16 L, and molecular weight is 476.4 kDa. PEM nephrotoxicity is well known; however, its frequency is considered to be low. The incidence of AKI reported by the Food and Drug Administration (FDA) is 2.4%. Renal damage ranges from acute to chronic kidney failure due to tubular and interstitial damage. Frequent association with other drugs, in particular cisplatin, explains the lack of more data on PEM nephrotoxicity (1-5).

Although the pathogenic mechanism of renal injury is not fully understood, a derangement between the PEM uptake carrier (folate receptor α, FR-α) and the basolateral transporters carrier (reduced folate carrier, RFC) of the proximal tubular cells has been suggested; thus, cytotoxicity is tied to an antifolate effect that impairs DNA and RNA (6).

Moreover, a proximal tubular damage, even if mild, as demonstrated in an experimental study published in 2005 (7), could increase drug nephrotoxicity, since the proximal tubule is the site where folic acid is reabsorbed through FR-α and RFC carriers. Besides, folate absorption is increased by an acidic environment, and decreased by a high urine flow, meaning that fluid and alkaline solutions administration could augment folate excretion, and as a consequence reduce its protective effects.

In the two cases we have reported above, the involvement of PEM in AKI development is quite clear. In the first case, the patient was given PEM in combination with NSAIDs, despite the well-known nephrotoxicity of both drugs, until the end of the sixth cycle. The patient developed renal failure only when the seventh cycle was administered and in the absence of other potential nephrotoxic agents. In the second case, although many AKI risks factors were present (dehydration, use of NSAIDs and radiocontrast agents), none of them seemed to be responsible for kidney injury. The lapse of time between the administration of radiocontrast agents and AKI development excludes radiocontrast agent induced nephropathy. NSAID consumption was inconsistent and suspension did not result in improvement. Moreover, kidney function did not improve when hypovolemia was corrected. However, we cannot exclude the contribution of these factors. We believe that risk factors for PEM-induced acute kidney damage include: 1) misleading GFR due to sarcopenia, 2) reduction in the total body water and hypoalbuminemia which can cause an increased drug concentration, 3) nephrotoxic combination drugs. In literature, kidney biopsies in patients with PEM-associated AKI show findings of acute tubular necrosis, tubular atrophy with chronic interstitial injury and acute interstitial nephritis. To our knowledge, the first patient described is the only case report with a big discrepancy between the severity of renal histopathological impairment and the degree of tubular damage. In fact, kidney biopsy showed only a modest tubular damage, confined to the proximal tubule, associated with mild interstitial edema. Nevertheless, the patient had only a modest recovery of renal function.

We reviewed the literature concerning kidney involvement during PEM therapy from 2003 up till now. We found 13 cases of proven renal involvement, including our own reports (Table 1). To the best of our knowledge, renal biopsy was performed only in 8 cases. Clinical and biochemical characteristics of the 13 patients are summarized in Table 1.

**Table 1:** Cases of renal involvement during PEM therapy reported in literature since 2003

**Ref.**	**Case No.**	**Age/Sex**	**Serum creatinine (mg/dL)**			**PEM cycles**	**Dose (mg/m** ^2^ **)**	**Combination chemotherapy**	**Nephrotoxic drugs**	**Renal biopsy**	**Biopsy findings**	**Tubular disfunction**	**Non-renal toxicity**	**Need for dialysis**
			**Baseline**	**Diagnosis**	**F-U**									
11	1	60/M	ND	3.1	ND	6	500	Cisplatin	Cisplatin	No	ND	No	Mucositis, Myelosuppression	Yes
8	1	53/F	0.8	4.1	1.7	3	500	-	Zometa, contrast media	No	ND	NDI, RTA	Myelosuppression	No
9	1	61/M	0.8	3.4	ND	2	500	Cisplatin	Cisplatin	No	ND	No	-	Yes
10	1	56/F	0.74	5.16	3.3	5	500	-	NSAID, contrast media	No	ND	No	Anemia	No
12	1	53/M	1.1	4.5	4.3	6	500	-	-	Yes	ATN+IF	No	-	No
13	1	57/M	0.94	3.4	HD	4	500	-	-	Yes	ATN+IF	No	-	Yes
6	1	67/M	0.9	2.6	2.1	-	500/39* 375/6*	-	ACE-i	Yes	ATI+TA+IF	Glicosuria	Anemia	No
	1	77/M	1.1	1.8	1.8	-	500/8* 375/6*	-	ARBs	Yes	ATI+TA+IF	No	-	No
	1	57/F	0.6	1.6	1.6	-	500/13*	-	ARBs	Yes	ATI+TA+IF	No	-	No
14	1	59/F	0.9	4.5	2.5	6	500	-	NSAID	Yes	ATN+IF	No	Edema of face	No
	1	60/M	1.0	4.0	2.46	4	500	Cisplatin	Cisplatin	Yes	ATN+IF	No	Edema of face	No
Current	1	52/M	1.0	6.7	4.1	7	500	-	NSAID	Yes	ATI	No	Anemia	Yes
	1	56/M	1.2	4.0	3	6	500	Carboplatin, taxol	Carboplatin	No	ND	No	Myelosuppression	No

Abbreviations: ATN, acute tubular necrosis; IF, interstitial fibrosis; IN, interstitial nephritis; ATI, acute tubular injury; TA, tubular atrophy; NSAID, non-steroidal anti-inflammatory drug; HD, hemodialysis; ND, no data; NDI, nephrogenic diabetes insipidus; RTA, renal tubular acidosis; ACE-i, angiotensin-converting-enzyme inhibitor; ARBs, angiotensin II receptor blockers. *mg/m^2^/no. of cycles.

Two cases of AKI reported in literature, showed renal insufficiency onset after three (8) and two (9) cycles of therapy, respectively. Both patients showed signs consistent with tubular dysfunction (diabetes insipidus and distal tubular acidosis). In a third case reported, AKI appeared after the sixth cycle (10). Castro et al (11) report a case of AKI after the sixth cycle. Unfortunately, none of these patients underwent kidney biopsy, so no data regarding histological features were provided.

Michels et al (12) and Stavroupoulos et al (13) published one case report each, in which both patients underwent kidney biopsy. The first developed acute tubular necrosis and interstitial nephritis after six cycles, the latter interstitial nephritis and diabetes insipidus after only four cycles.

Glezerman et al (6) describes three patients who developed chronic kidney disease while undergoing maintenance therapy with PEM. Kidney biopsy specimens showed tubulointerstitial injury with tubular simplification, shrinkage, and tubular atrophy. Kidney function remained impaired, but stable, after discontinuation of PEM therapy. The first patient described showed appearance of glycosuria caused by tubular damage.

Chauvet et al (14) report two cases of AKI with biopsy-proven renal tubular toxicity. Renal toxicity in both patients was associated with face edema, a rare side effect of the drug.

Among all cases reported in literature, only 4 patients required hemodialysis, but functional recovery was partial in all of them, even months after drug discontinuation, as was also the case with the two patients we are reporting here.

The therapy of PEM toxicity is not clear. Castro et al (11) suggested use of thymidine as a rescue therapy in a patient who developed acute PEM intoxication after the sixth cycle, but its efficacy on renal damage has not been proven. In patients with AKI, based on PEM pharmacokinetic characteristics, Rombolà et al (15) suggested prolonged dialysis to reduce renal toxicity and length of hospitalization, although the effect of this has not been studied.

## 4. Conclusions

In conclusion, we would like to raise the following caution. Although the landscape of NSCLC has changed by the availability of PEM, the risk of acute or chronic kidney disease may be one of the prices to be paid for this success. The use of creatinine-based estimations may be significantly affected by creatinine generation, specifically in the oncologic population with decreased muscle mass. Consequently, we underline that estimated GFR (eGFR) in oncologic patients could be overrated, thus we strongly advise to carefully monitor renal function, also in presence of unremarkable urinary sediment. The measurement of renal function in these patients should be validated using a more specific non-creatinine based assay. Moreover, it is also mandatory to avoid potential nephrotoxic agents, such as NSAIDs and radiocontrast agents, and to correct all causes that reduce effective intravascular volume (vomiting, diarrhea, sepsis, ascites, congestive heart failure, hypoalbuminemia, etc), in order to prevent renal injury. Thus, nephrologists and oncologists should collaborate to study tailored therapies in oncologic patients.

## Authors’ contribution

ZT, LF, FV, RD, CN, CM; wrote the manuscript. CV, PR, TP; performed histological investigation. TM, GA, ST; carried out ultrasonographic examination. ML, AM; revised English version. All authors run equally literature review.

## Conflicts of interest

The authors declare that they have no conflicting interest.

## Funding/Support

None.
